# Spatiotemporal control of l-phenyl­alanine crystallization in microemulsion: the role of water in mediating molecular self-assembly

**DOI:** 10.1107/S2052252522003001

**Published:** 2022-04-27

**Authors:** Qi Liu, Jingkang Wang, Xin Huang, Hao Wu, Shuyi Zong, Xiaowei Cheng, Hongxun Hao

**Affiliations:** a National Engineering Research Center of Industrial Crystallization Technology, School of Chemical Engineering and Technology, Tianjin University, Tianjin 300072, People’s Republic of China; b State Key Laboratory of Chemical Engineering, Tianjin University, Tianjin 300072, People’s Republic of China; c School of Chemical Engineering and Technology, Hainan University, Haikou 570228, People’s Republic of China

**Keywords:** l-phenyl­alanine crystallization, microemulsion nanoconfinement, amyloid fibril, self-assembly, morphology, crystal engineering, crystal morphology, polymorphism, pharmaceutical solids

## Abstract

Water confined or constrained in a cellular environment can exhibit a diverse structural and dynamical role and hence will affect the self-assembly behavior of biomolecules. Herein, the role of water in the formation of l-phenyl­alanine crystals and amyloid fibrils was investigated. A microemulsion biomimetic system with controllable water pool size was employed to provide a microenvironment with different types of water, which was characterized by small-angle X-ray scattering, attenuated total reflectance-Fourier transform infrared spectroscopy and differential scanning calorimetry. In a bound water environment, only plate-like l-phenyl­alanine crystals and their aggregates were formed, all of which are anhydrous crystal form I. However, when free water dominated, amyloid fibrils were observed. Free water not only stabilizes new oligomers in the initial nucleation stage but also forms bridged hydrogen bonds to induce vertical stacking to form a fibrous structure. The conformational changes of l-phenyl­alanine in different environments were detected by NMR. Different types of water trigger different nucleation and growth pathways, providing a new perspective for understanding molecular self-assembly in nanoconfinement.

## Abstract 2

In nanoconfinement environments, different types of water trigger different forms of self-assembly of high-quality l-phenyl­alanine crystals through molecular oriented attachment. In addition, it was also found that the formation of l-phenyl­alanine amyloid fibrils is related to free water.

## 1. Introduction

Confinement of aqueous solution is ubiquitous in biological systems, water plays an active and complex role in the structure, stability, dynamics and function of biomolecules, *e.g.* the process of protein folding or fibrosis (Levy & Onuchic, 2006 ▸; Ball, 2017 ▸). Inspired by biology, W/O microemulsions (MEs) have been employed frequently as biomimetic systems (Hayes, 2014 ▸). Owing to its unique properties, which include transparency, thermodynamic stability, confinement within soft interfaces and together with its abilities to solubilize various guest molecules, ME was suggested as an ideal matrix for crystallization. Water in extreme confinement has different physicochemical properties than in bulk (Levinger, 2002 ▸). Not surprisingly, the dynamics of nanoscopic water along with its structure have attracted widespread attention (Moilanen *et al.*, 2007 ▸; Salvati Manni *et al.*, 2019 ▸). The interior of a confinement system exists as at least two regions corresponding to interfacial ‘bound water’ and central ‘free water’. The proportion of different types of water in ME can be controlled by adjusting the water content (Aggrawal *et al.*, 2020 ▸). In this regard, whether the water state has an important effect on crystallization or self-assembly of small biomolecules in ME has aroused great interest. This strategy has positive significance in fields as diverse as pharmaceuticals, nanomaterial synthesis, protein crystallography and biomineralization (Landau & Rosenbusch, 1996 ▸; Nicholson *et al.*, 2011 ▸; Lu *et al.*, 2021 ▸; Meldrum & O’Shaughnessy, 2020 ▸).

l-phenyl­alanine (l-Phe) is an essential amino acid for the human body, its crystallography and biological functions have always attracted attention (Ihlefeldt *et al.*, 2014 ▸; Adler-Abramovich *et al.*, 2012 ▸). l-Phe monohydrate is more stable below 37°C in aqueous solution (Wang *et al.*, 2014 ▸). Crystallization of l-Phe itself is not trivial, Khawas (1970 ▸) noted that that it ‘could not be obtained as good single crystals by the ordinary methods of crystallization’. Besides, Adler-Abramovich *et al.* (2012 ▸) first demonstrated that a single l-Phe can self-assemble into amyloid fibrils, which would cause phenyl­ketonuria (PKU). Some investigators have already attempted to reveal the self-assembly mechanism of l-Phe fibrils, expecting to provide effective guidance for the therapy of PKU (Banerjee *et al.*, 2020 ▸; Singh *et al.*, 2014 ▸). It is generally believed that hydrogen bonding, hydro­phobic interaction or electrostatic interaction between l-Phe molecules is responsible for the formation of amyloid fibrils, and efforts have been made to treat PKU by inhibiting fibril formation (Tomar *et al.*, 2019 ▸; Banik *et al.*, 2016 ▸; Do *et al.*, 2015 ▸; German *et al.*, 2015 ▸; Singh *et al.*, 2014 ▸). However, the above phenomena will be different when l-Phe is in the ME, including polymorph stability, crystal quality and inhibition of amyloid fibrils formation. The fundamental crystallization mechanism in confinement systems is still not understood, but the water state is believed to play a critical role in nucleation and crystal growth (Vallooran *et al.*, 2019 ▸).

## 2. Experimental

### 2.1. Materials

l-Phe (purity ≥ 99%), cyclo­hexane (purity ≥ 99.5%) and 1-pentanol (purity ≥ 99%) were purchased from Shanghai Macklin Biochemical Technology Co. Ltd. Triton X-100 (purity ≥ 99%, FW = 674) was purchased from Tianjin Dingguo Biotechnology Co. Ltd. Chloro­form-*d* (99.8 + 0.03%TMS) was purchased from Tianjin heowns Biochemical Technology Co. Ltd. All materials were used without further purification. Deionized water was prepared by the Millipore water system with a resistivity of 18.2 MΩ cm.

### 2.2. Sample preparation in microemulsion

l-Phe was dissolved in water at 55°C to prepare the solutions with the concentrations 120, 150, 180 and 210 m*M*. Then, the solutions were cooled to 35°C and maintained for 1 h. A mass of 10 g stock solution with a fixed mass ratio of cyclo­hexane:(Triton X-100):(1-pentanol) = 4:3:1 was prepared and kept at 35°C. The ME was formed by mixing the stock solution with 0.1, 0.3, 0.5, 1 and 2 ml l-Phe aqueous solution (corresponding to *w*_o_ = 1, 3, 5, 10, 20) using an ultrasonic dispersing processor (UP-400S, Jingxin) at 35°C, followed by incubation at 15°C. After 5 days, the sample was transferred to an incubator for a extended incubation and used for further analysis.

### 2.3. Solubility test of l-phenyl­alanine in microemulsion

A small amount of l-Phe powder (1–3 mg) was added to the prepared ME and stirred at 400 rpm until l-Phe completely dissolved. This procedure was repeated until l-Phe no longer dissolved. Then, the saturated l-Phe ME was maintained for one month in a constant-temperature incubator; no occurrence of crystallization was confirmed by microscopy observation during the storage period. The total added mass of l-Phe was used to calculate its solubility in the ME.

### 2.4. l-Phenyl­alanine monohydrate preparation

The excess l-Phe was dissolved in water at 55°C by magnetic stirring, and the supernatant was removed after stirring for 4 h. The supernatant collected was maintained at 60°C for 2 h, then transferred to a 10°C water bath for rapid cooling and incubated for 12 h. Next, the temperature was raised to 15°C at 5 K h^−1^ and maintained for 2 h, then cooled to 10°C at 5 K h^−1^ and maintained for 4 h. Finally, the sample was filtered and dried at 30°C under vacuum. The polymorph of the sample was determined to be the l-Phe monohydrate by XRD, as shown in Fig. S8 of the supporting information.

### 2.5. Sample collection

The ME containing l-Phe solids was centrifuged at 12 000 rpm for 10 min to collect the supernatant (3-18KS, SIGMA). The supernatant was further centrifuged again for 30 min and the pure supernatant obtained was used for subsequent small-angle X-ray scattering (SAXS), attenuated total reflectance-Fourier transform infrared spectroscopy (ATR-FTIR), nuclear magnetic resonance spectroscopy (NMR) and differential scanning calorimetry (DSC) analyses. The precipitate was dispersed in 1-pentanol for centrifugation, which was repeated twice. Similarly, the precipitate collected was further dispersed in cyclo­hexane, and was centrifuged twice again. Finally, the solid samples were obtained by freeze-drying and used for subsequent scanning electron microscopy (SEM) and X-ray diffraction (XRD) analyses.

### 2.6. Polarized light microscopy

A drop of the ME mixture containing l-Phe solid was added onto a glass slide and covered for observation.

### 2.7. Scanning electron microscopy

The sample was laid on the conductive tape and coated with gold and the morphology of l-Phe fibril was analyzed by SEM (TM3000, Hitachi).

### 2.8. Transmission electron microscopy

The sample was prepared by dropping ME containing l-Phe fibril onto 400-mesh copper grids, then the ME was removed. Subsequently, 1-pentanol and cyclo­hexane were dripped to clean the fibril. Finally, the sample was dried at room temperature. The morphology of the l-Phe fibril was analyzed by transmission electron microscopy (TEM) (FEI Tecnai G2 F20). Selected-area electron diffraction (SAED) analysis was carried out during high-resolution TEM.

### 2.9. Small-angle X-ray scattering

SAXS profiles were collected using SAXSpace (Anton Paar) at 50 kV/1 mA with a Cu *K*α radiation source (λ = 1.542 Å). The ME sample was sealed in a TCS capillary holder, which was mounted on a TCStage test platform and measured at 15°C.

### 2.10. X-ray diffraction

XRD data were collected on a D/max-2500 diffractometer (Rigaku) at 40 kV/100 mA with a Cu *K*α radiation source (λ = 1.54056 Å). The samples were scanned from 2 to 40° (2θ) at a step size of 0.02° and a scanning rate of 8° min^−1^.

### 2.11. Laser confocal Raman spectrometer

The ME mixture containing l-Phe fibrils was dropped on a glass slide and covered. The samples were characterized using a Renishaw inVia Reflex Raman microscope at an excitation wavelength of 532 nm with a spectral resolution of ∼1 cm^−1^.

### 2.12. Attenuated total reflectance-Fourier transform infrared spectroscopy

The samples were characterized by ATR-FTIR (α, Bruker) with a resolution of 2 cm^−1^ and wavenumber ranging from 400 to 4000 cm^−1^.

### 2.13. Nuclear magnetic resonance spectroscopy

A series of ^1^H solution-state NMR experiments were performed using a Bruker Avance III 600 MHz spectrometer. The ME sample was placed in an external nuclear magnetic tube, and CDCl_3_ (containing TMS) was placed in a coaxial inner cell as an external lock and reference solvent.

### 2.14. Differential scanning calorimetry

The experiments were performed on a TA DSC Q2000 and carried out with an empty sample pan as the reference. The samples were sealed and loaded in aluminium crucibles and then scanned for the temperature range from 25 to −75°C. First, the temperature was maintained at 25°C for 10 min, and then cooled from 25 to −75°C at a rate of 30°C min^−1^. Then the temperature was held constant again for 10 min, and finally heated from −75 to 25°C at a rate of 3°C min^−1^.

## 3. Results and discussion

**Figure 1:**
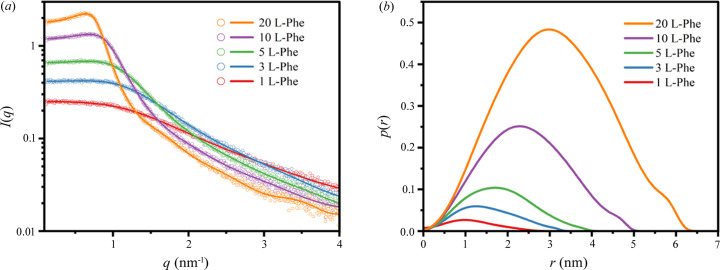
(*a*) 1D SAXS profiles of ME with increasing l-Phe aqueous solution content (*w*_o_ = 1, 3, 5, 10, 20), the circles represent the experimental data and the solid line is the GIFT fit. (*b*) PDDF of the ME; the corresponding radii are 1.22, 1.54, 1.81, 2.39 and 3.07 nm.

In previous work, we successfully developed a model system of water/Triton X-100/1-pentanol/cyclo­hexane to study the glycine self-assembly process (Liu *et al.*, 2020 ▸). The system contains a fixed mass ratio of cyclo­hexane:(Triton X-100):(1-pentanol) = 4:3:1, which is then mixed with a certain amount of l-Phe aqueous solution (or water). Herein, the molar ratio *w*_o_ = [water]/[Triton X-100] can be defined and a series of MEs with different *w*_o_ values were obtained by changing the water content. For the ME structure, SAXS profiles of the ME analyzed by the generalized indirect Fourier transformation technique (GIFT) (Fritz & Glatter, 2006 ▸; De Campo *et al.*, 2004 ▸; Guinier & Fournet, 1955 ▸) were adopted to give highly approximate results [Fig. 1 ▸(*a*)]. The corresponding ME structure information can be obtained by the pair-distance distribution functions (PDDFs), *p*(*r*), shown in Fig. 1 ▸(*b*). The results indicate that the shape of the ME droplets is approximately spheroid, and the radius increases monotonically from 1.22 to 3.07 nm with increasing *w*_o_. Here, we successfully obtained matrix of the ME with a nanoconfined structure.

**Figure 2:**
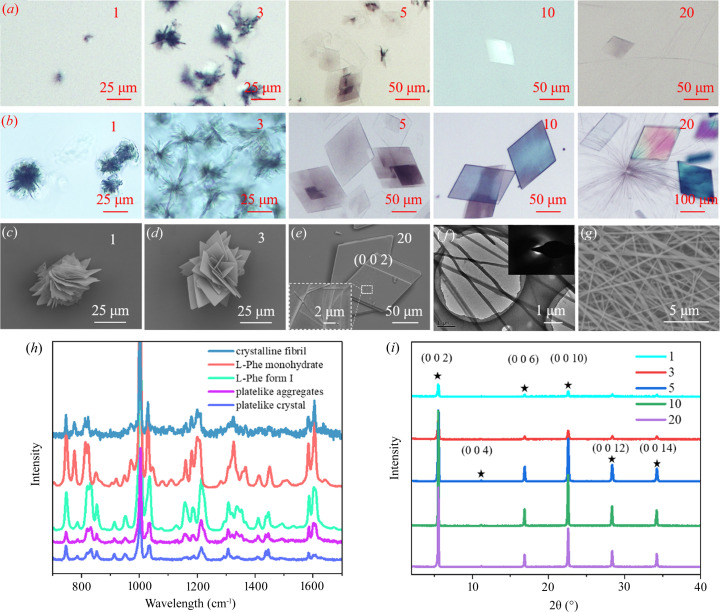
Optical microscopy of l-Phe after (*a*) 4 h and (*b*) 3 days (*w*_o_ = 1, 3, 5, 10, 20; 210 m*M* l-Phe aqueous solution). SEM image of l-Phe with (*c*) *w*_o_ = 1, (*d*) *w*_o_ = 3 and (*e*) *w*_o_ = 20; the inset shows an enlarged view of the fibril structure. (*f*) TEM image of l-Phe in ME with fibrils, the inset shows the SAED pattern. (*g*) TEM image of the crystalline fibril structure. (*h*) Laser confocal Raman profiles of l-Phe with different morphology, reference spectra of l-Phe monohydrate and anhydrate. (*i*) XRD patterns of l-Phe with different *w*_o_values.

Nanoconfinement often influences the molecule nucleation and crystal growth (Meldrum & O’Shaughnessy, 2020 ▸). A freshly prepared l-Phe-ME (210 m*M*) was incubated at 15°C (see sample preparation of the ME in the experimental[Sec sec2] for details) and the l-Phe solids can be clearly observed after 4 h [Fig. 2 ▸(*a*)]. When the incubation was prolonged to 3 days, the morphology of l-Phe tended to be stable [Fig. 2 ▸(*b*)]. The compact flake aggregates transformed into plate-like aggregates when the water content increased from *w*_o_ = 1 to *w*_o_ = 3 and then into plate-like crystals when *w*_o_ = 5, 10 and 20, as shown in the SEM images [Figs. 2 ▸(*c*)–2 ▸(*e*)]. Note that a small fibril also appeared when *w*_o_ = 20. In fact, the fibrosis process began when the l-Phe aqueous solution was prepared (Banerjee *et al.*, 2020 ▸). It is clear the formation of the l-Phe fibril could be inhibited in ME. The fibril was characterized by TEM and no diffraction point was found in the SAED pattern [Fig. 2 ▸(*f*)], which is consistent with reported results (Banerjee *et al.*, 2020 ▸). The structure of l-Phe with different morphologies was analyzed by laser confocal Raman spectroscopy [Fig. 2 ▸(*h*)], the results indicated that both the plate-like aggregates and crystals were l-Phe form I (anhydrate) (Zhu *et al.*, 2011 ▸); unfortunately, the fibril was too thin to obtain a spectrum. However, we found that the fibrils crystallized gradually after two months of incubation [Fig. 2 ▸(*g*) and Fig. S1] and exhibit the characteristics of l-Phe monohydrate [Fig. 2 ▸(*h*)]. In addition, the structure of l-Phe was further analyzed by XRD [Fig. 2 ▸(*i*)], the diffraction peaks of [002] are in accordance with l-Phe form I, ranging from *w*_o_ = 1 to *w*_o_ = 20. XRD did not detect the fibril structure, possibly because the fiber is amorphous or the content was too small.

**Figure 3:**
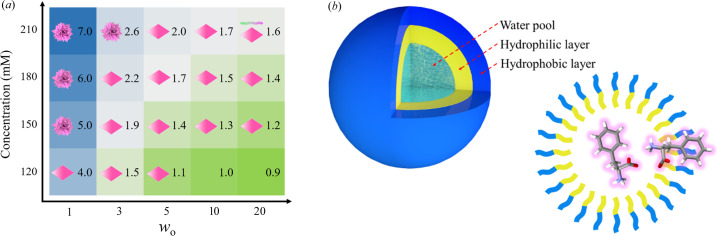
(*a*) Morphology of l-Phe at different concentrations and contents of l-Phe aqueous solution in the ME, and supersaturation is also given in the corresponding table cell. (*b*) Schematic of the structure of the ME, l-Phe is in the water pool and the interface of the ME.

Moreover, the crystallization of l-Phe at different concentrations was investigated (Fig. S2), and the solubility of l-Phe in the ME was also measured (Fig. S3). Based on the above experimental results, a coordinate system about *w*_o_ and concentration was established and the morphologies of l-Phe as well as the corresponding supersaturation were filled [Fig. 3 ▸(*a*)]. l-Phe aggregates and plate-like crystals were observed in a wide range of supersaturation (*S* = 1.0–7.0) and *w*_o_ (*w*_o_ = 1–20). Fibrils form when *w*_o_ is large enough or higher than a certain value, even in the lower supersaturation situation (*S* = 1.6, *w*_o_ = 20). The polymorph of l-Phe is mainly related to *w*_o_, followed by supersaturation. The solvent effect of water on l-Phe is understandable, it is also reflected in that the solubility of l-Phe in ME increases with *w*_o_, and eventually exceeds the solubility in bulk water (Fig. S3). This is because l-Phe has a lower solubility in bound water than free water and can be solubilized at the interface due to its amphiphilic nature [Fig. 3 ▸(*b*)] (Leodidis & Hatton, 1990*a* ▸,*b* ▸; Adachi *et al.*, 1991 ▸). The change in the type of water has an important impact on the self-assembly behavior of l-Phe.

**Figure 4:**
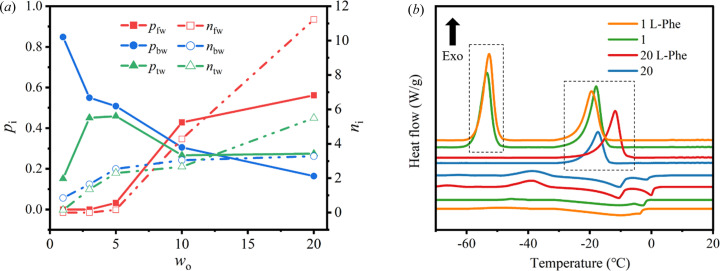
(*a*) Proportion (*p*_i_) and number (*n*_i_) of free water (i = fw), bound water (i = bw) and trapped water (i = tw) molecules in the ME vary with *w*_o_; *n*_i_ refers to the number of water molecules per molecule of surfactant. (*b*) DSC thermograms of the ME with and without l-Phe.

In order to analyze the solvent water, ATR-FTIR was used to distinguish the types of water in the ME (Zhao *et al.*, 2007 ▸; Brubach *et al.*, 2001 ▸; Yuan *et al.*, 2004 ▸). The O—H stretching vibrations region (3000–3700 cm^−1^) in ATR-FTIR profiles of the ME was deconvoluted as sums of single-peaked Gaussian distributions (Fig. S4). The results indicate that there are three types of water: free water, bound water and trapped water. Moreover, the integrals of the fitted function, the proportion (*p*_i_) and number (*n*_i_) of the types of water in the ME varying with *w*_o_ are given in Fig. 4 ▸(*a*). It has been found that free water appears in large quantities when *w*_o_ = 10, and bound water content tends to stabilize (Andrade *et al.*, 2000 ▸; Robson & Dennis, 1977 ▸). The presence of water molecules in the l-Phe fibril has been confirmed (Singh *et al.*, 2017 ▸). When free water increases to a certain degree, it will participate in the self-assembly of l-Phe and eventually lead to fibril formation. It is reasonable to infer that hydration plays an important role in molecular self-assembly (Pal & Zewail, 2004 ▸). This aspect was further investigated by DSC (Vallooran *et al.*, 2019 ▸) and the results are shown in Fig. 4 ▸(*b*). There are two exothermic peaks which correspond to bound water freezing (about −53°C) and cyclo­hexane freezing (about −18°C) when *w*_o_ = 1. When *w*_o_ = 20, exothermic peaks corresponding to cyclo­hexane and water freezing merge into one peak. The water state shows variation under different *w*_o_. Note that the freezing point of the ME with or without l-Phe varies greatly from −11.7°C to −17.5°C when free water dominates (*w*_o_ = 20). This implies that l-Phe is involved in the composition of the ME and may cause conformational changes.

**Figure 5:**
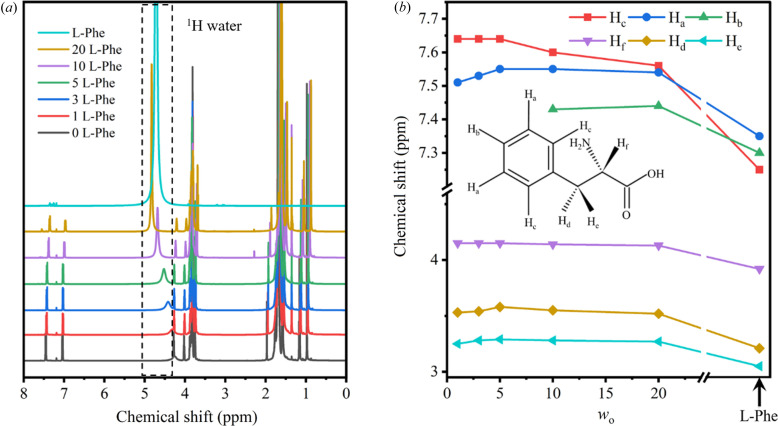
(*a*) ^1^H NMR spectra of l-Phe-ME with different *w*_o_ and l-Phe bulk solution (l-Phe), the dashed box is ^1^H of water. (*b*) Change trend of ^1^H NMR of l-Phe with increasing *w*_o_, the arrow indicates l-Phe bulk solution. Due to the masking of Triton X-100, H_b_ is absent at *w*_o_ = 1, 3, 5.

Next, the microenvironment of l-Phe-ME was studied by NMR, which is sensitive to species interactions and conformational changes. The ^1^H NMR chemical shift (δ) of water displays a downfield shift as *w*_o_ increases [Figs. 5 ▸(*a*) and S5(*a*)], which pertains to the electron density around the proton and reflects the change of the local property of the water molecules (Waysbort *et al.*, 1997 ▸). The chemical shift of ^1^H water is closer to that of bulk water at high *w*_o_, corresponding to the results of ATR-FTIR and DSC. The state of l-Phe was also examined [Figs. 5 ▸(*b*), S5 and S6]. Chemical shifts of aromatic protons in bound water (*w*_o_ = 1, δ_c_ > δ_a_ > δ_b_) and bulk water (δ_a_ > δ_b_ > δ_c_) vary greatly. In particular δ_c_ significantly decreased and approached δ_a_ gradually when *w*_o_ increased, indicating a conformational change of l-Phe. Overall, l-Phe can form more hydrogen bonds and weaken the shielding effect as the proportion of bound water decreases, leading to an increase in chemical shift; this is more obvious at low *w*_o_ (1 < *w*_o_ < 5). However, owing to conformational changes, the chemical shift eventually slightly decreased at high *w*_o_ (5 < *w*_o_ < 20). We conclude that l-Phe has different conformations in bound water and free water.

**Figure 6:**
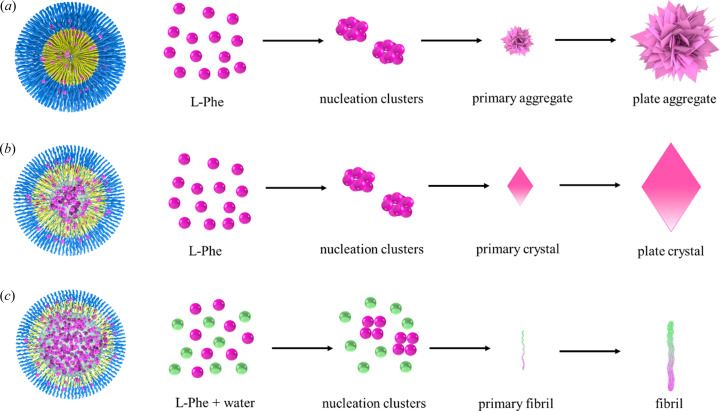
Dynamic evolution process of l-Phe self-assembly in the ME of (*a*) aggregates, (*b*) plate-like crystals and (*c*) fibrils with increasing water content (or *w*_o_).

The dynamic evolution process of l-Phe self-assembly in the ME was summarized (Fig. 6 ▸). In an environment with bound water around the interface of the ME, water molecules are tightly bound to surfactants rather than l-Phe. Thus, l-Phe tends to form an anhydrate (form I) instead of a monohydrate, although the monohydrate precipitates in bulk water (<37°C). The smaller the ME size, the more stable l-Phe is at the interface. It is further considered that the ME contains fewer l-Phe molecules, hence a stronger driving force for crystallization is required. Burst nucleation occurs easily under higher saturation, and then aggregative growth [Fig. 6 ▸(*a*)]. When the size of the ME increases or the saturation is low, the nucleation rate slows down, and aggregation does not occur [Fig. 6 ▸(*b*)]. When the size of the ME is further increased to the extent that free water dominates, l-Phe in free water will self-assemble into amyloid fibrils [Fig. 6 ▸(*c*)]. Amyloid fibrils are formed at high concentrations as a result of competition between the thermodynamically controlled fibrillation process in the water pool and the dynamically controlled crystallization process at the interface. Banerjee *et al.* (2020 ▸) observed the phenomenon of fibril-to-crystal conversion through evaporation of bulk solution; however, the limitations of the method make the conversion incomplete. The ME provides a long-term incubation environment for the fibril-to-crystal conversion to finally obtain the l-Phe monohydrate, where water molecules are evidently involved in this process.

**Figure 7:**
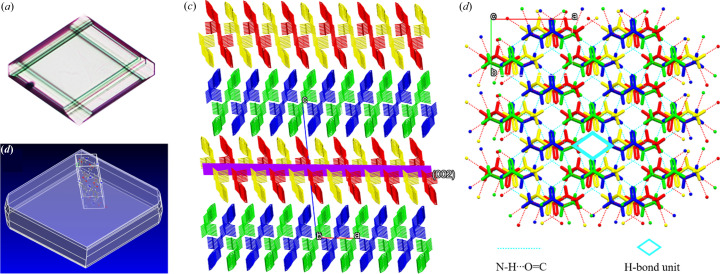
(*a*) Optical micrograph of plate-like l-Phe. (*b*) Equilibrium morphology calculation of l-Phe form I in *Materials Studio* (version 6.0; Gibbs, 1929 ▸), (*c*) Crystal structure of form I, viewed along the *b* axis. (*d*) Hydrogen-bonded network of form I, viewed along the *c* axis.

Finally, a possible mechanism for the morphology and structure of l-Phe was proposed. For plate-like crystals, l-Phe is located at the interface of the ME and has an unfolded conformation: aliphatic motif outward and aromatic motif inward. It is more conducive to oriented attachment during the ME droplet collision, forming rhombus-like crystals with a layered structure [Fig. 7 ▸(*a*)]. In Fig. 7 ▸(*b*), for the morphology prediction which considers that, in equilibrium, the crystal habit minimizes the surface energy (Gibbs, 1929 ▸), the results match those of the experiments. In Fig. 7 ▸(*c*), two antiparallel β-sheets produce a β-strand, two different β-strands consecutively repeat throughout the crystal packing by edge-to-face π−π stacking interactions between hydro­phobic phenyl rings (Bera *et al.*, 2018 ▸). The (002) plane maintains the integrity of l-Phe form I: there are rhombus-like hydrogen-bonded units in the hydrogen-bonded network viewed along the *c* axis [Fig. 7 ▸(*d*)]. l-Phe crystallizes in its natural form; the macromorphology is consistent with the crystal structure. 2D crystals obtained in nanoconfinement are uncommon, this is an effective method for obtaining high-quality crystals (Landau & Rosenbusch, 1996 ▸). For amyloid fibrils, l-Phe is more flexible in free water and forms new molecular clusters; from previously reports, it was considered to be a tubular structure of the l-Phe tetramer stacked in layers with a maximum pore diameter of 5 Å by a molecular dynamics study (Fig. S7) (German *et al.*, 2015 ▸). Although water molecules can move in the channel of l-Phe monohydrate (Williams *et al.*, 2013 ▸), it is speculated that water channel exists in the fibril structure, as supported by other similar reports (Wang *et al.*, 2017 ▸). Free water in the channel could hydrate the l-Phe polar side chains and induce fibril formation through the bridged hydrogen bond of water and the central hydro­philic core of clusters. The contribution of hydro­phobic or van der Waals interactions in fibrillation also cannot be ignored (Singh *et al.*, 2014 ▸). With aging, water molecules finally become a part of the fibril due to thermodynamic stability and l-Phe monohydrate is produced. The process of fibrillation in ME is elongated compared with in bulk solution, and the important role of water molecules in fibrillation is better understood.

## 4. Conclusions

We systematically studied the behaviors of l-Phe in the W/O ME biomimetic system. The compartmentation of l-Phe slows down the crystallization or self-assembly process, making it easier to obtain high-quality crystals and to study fibrillation. This work utilized the characteristics of the different types of water in the ME to realize control of the structures and morphologies. l-Phe around the interface cannot use bound water, forming anhydrate form I. Besides, changing the conformation of l-Phe or hindering the bridging of water in l-Phe cluster stacking will inhibit the formation of fibrils, which provides an effective strategy for the therapeutic treatment of PKU. These findings demonstrate the key role of water in the hierarchical crystallization and self-assembly of biomolecules in the nanoconfinement environment, which can be applied to construct advanced functional materials for nanotechnology and biomedicine, and provides a platform for understanding amyloid fibrils.

## Supplementary Material

Supporting figures. DOI: 10.1107/S2052252522003001/ed5025sup1.pdf
