# Supplementary material for: Spatiotemporal control of l-phenyl­alanine crystallization in microemulsion: the role of water in mediating molecular self-assembly
Source: IUCrJ. 2022 Apr 27;9(Pt 3):370–7. doi: 10.1107/S2052252522003001 (PMC9067117; doi:10.1107/S2052252522003001)
Supplement: Supplementary file 1 [file m-09-00370-sup1.pdf]

IUCrJ

Volume 9 (2022)

Supporting information for article:

Spatiotemporal control of l-phenylalanine crystallization in microemulsion: the role of water in mediating molecular self-assembly

Qi Liu, Jingkang Wang, Xin Huang, Hao Wu, Shuyi Zong, Xiaowei Cheng and Hongxun Hao

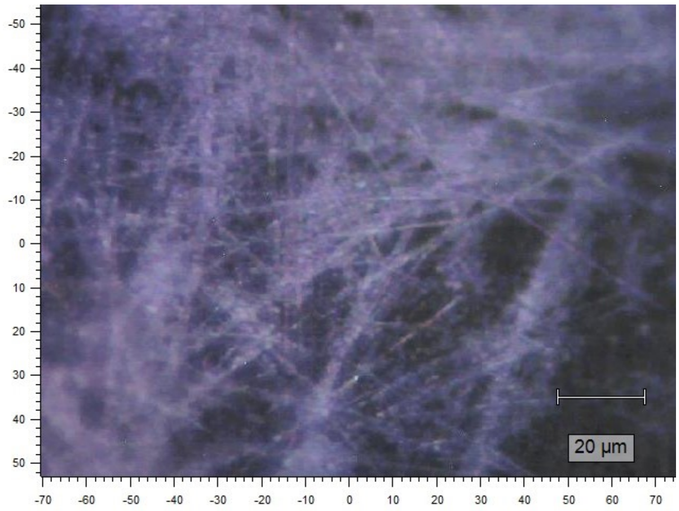

Figure S1 The images of crystalline fibril structure measured by laser confocal Raman spectroscopy.

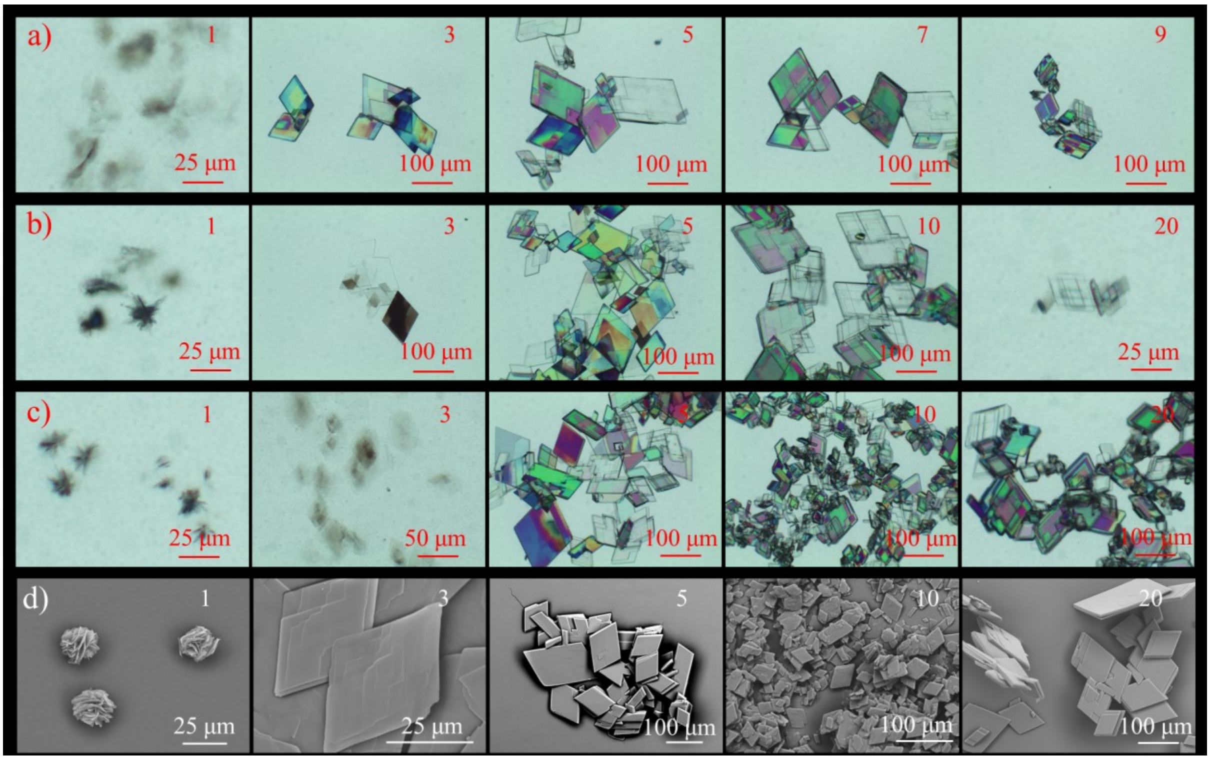

Figure S2 Optical micrograph of L-Phe with different w_o at concentration of a) 120 mM, b) 150 mM, c) 180 mM. d) SEM images of L-Phe at concentration of 180 mM. w_o is on the top right of each image.

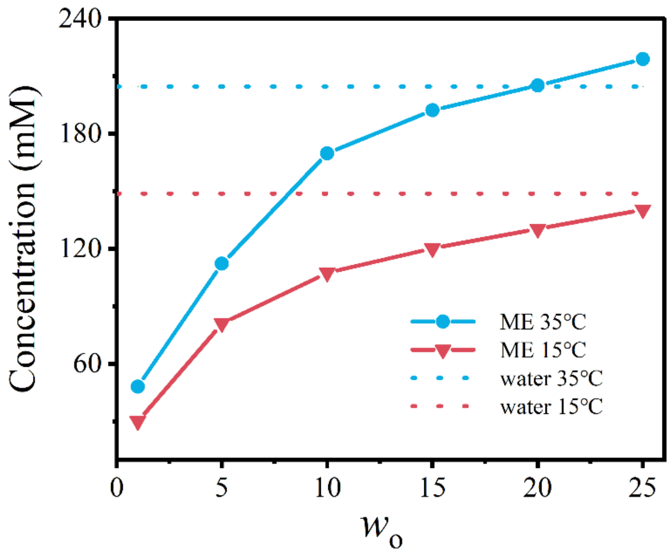

Figure S3 Solubility of L-Phe in microemulsion at 15 °C and 35 °C. The dotted line represents the solubility of L-Phe in pure water, and the solid line represents the ratio of L-Phe in water of ME.

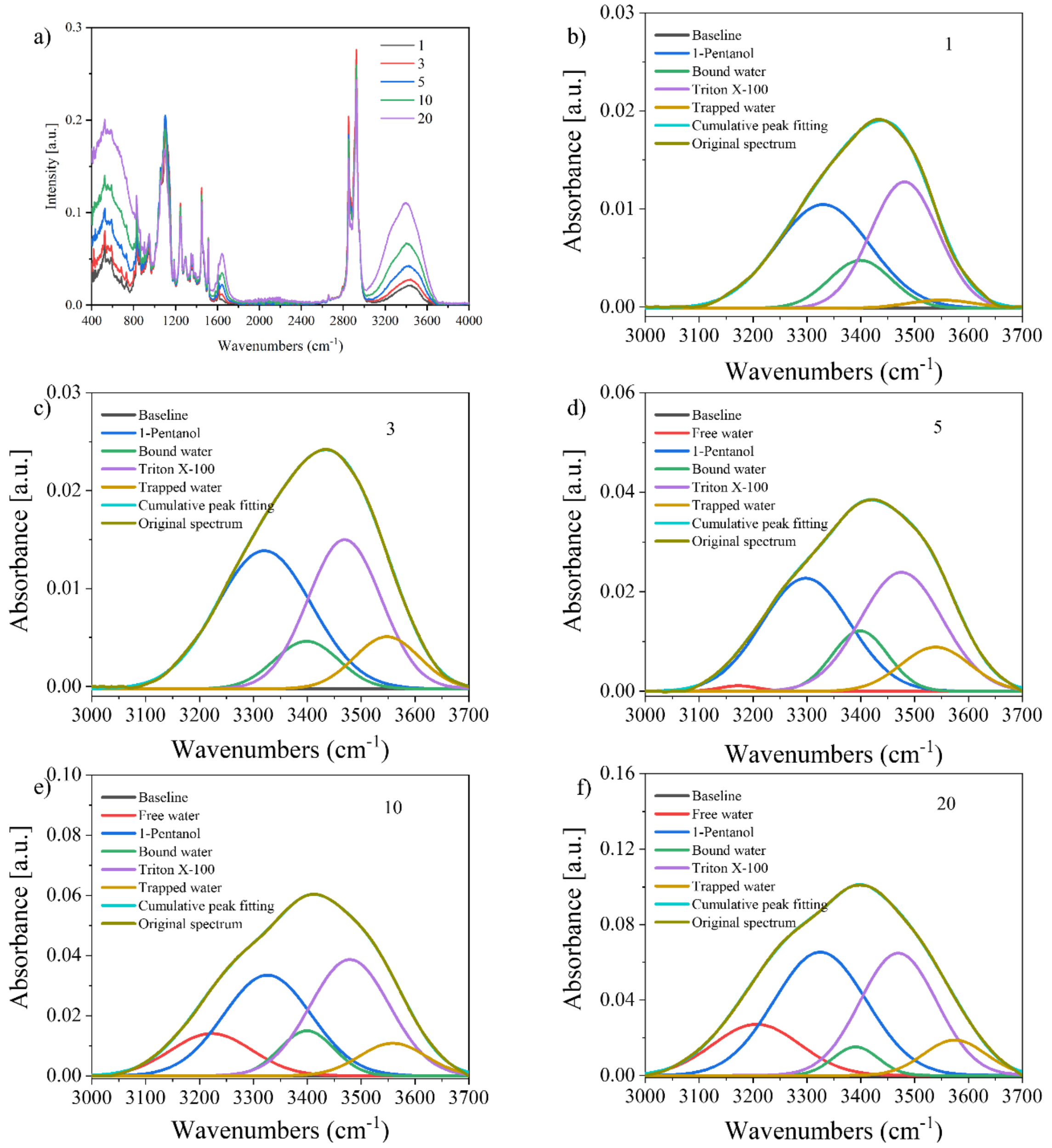

Figure S4 a) ATR-FTIR spectra of ME (w_o = 1, 3, 5, 10, 20). b-f) The O–H stretching region (3000–3700 cm^{-1}) of ME in all systems were fitted with a Gaussian program in OriginLab software, the fitting peaks correspond to free water (3200 ± 20), 1-pentanol (3310 ± 10), bound water (3395 ± 10), Triton X-100 (3470 ± 10), trapped water (3555 ± 10), respectively.

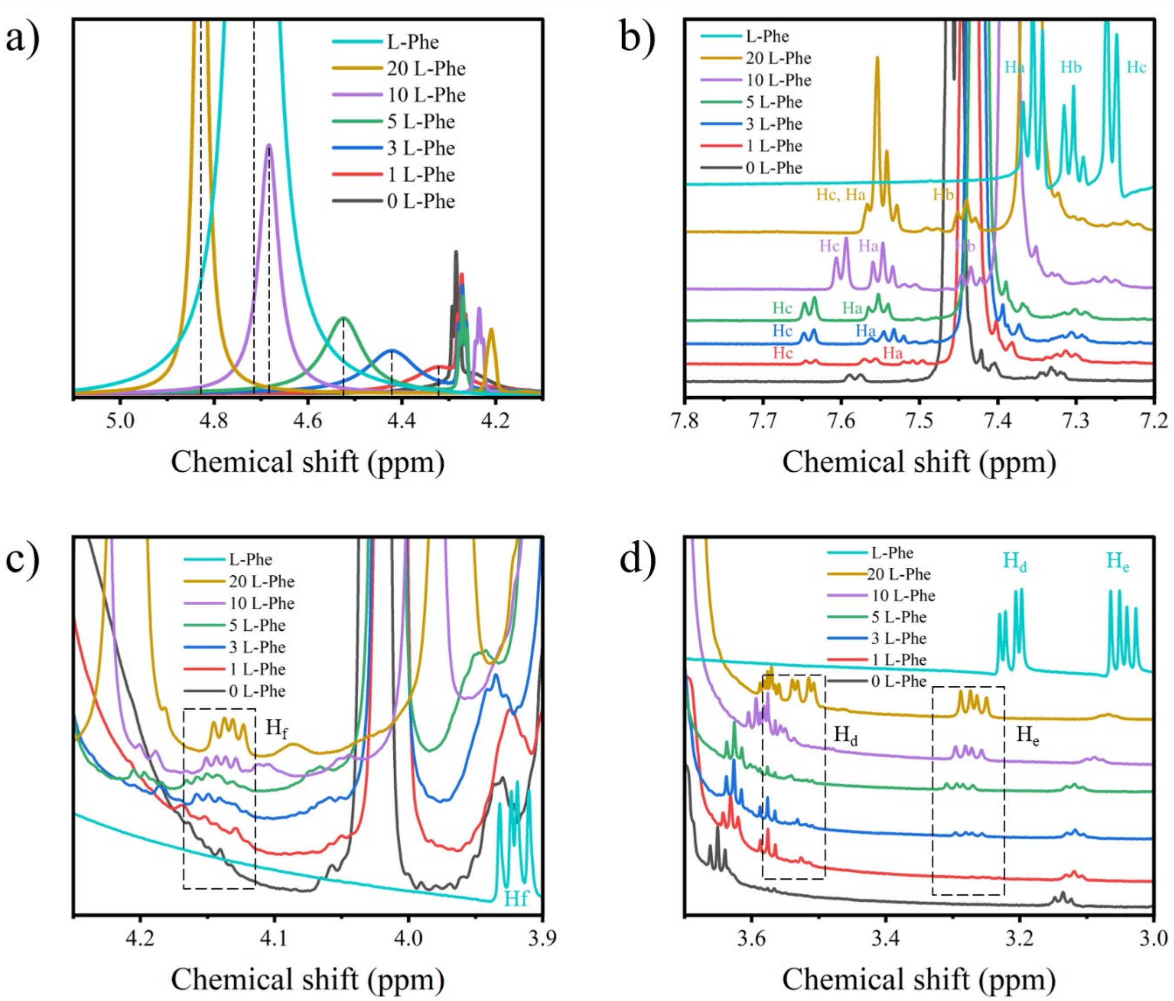

Figure S5 ^{1}H NMR profiles of L-Phe in microemulsion. Partial enlarged drawing in Figure 4a corresponds to a) ^{1}H of water, b) H_a, H_b, H_c of L-Phe, c) H_f of L-Phe, d) H_d of L-Phe.

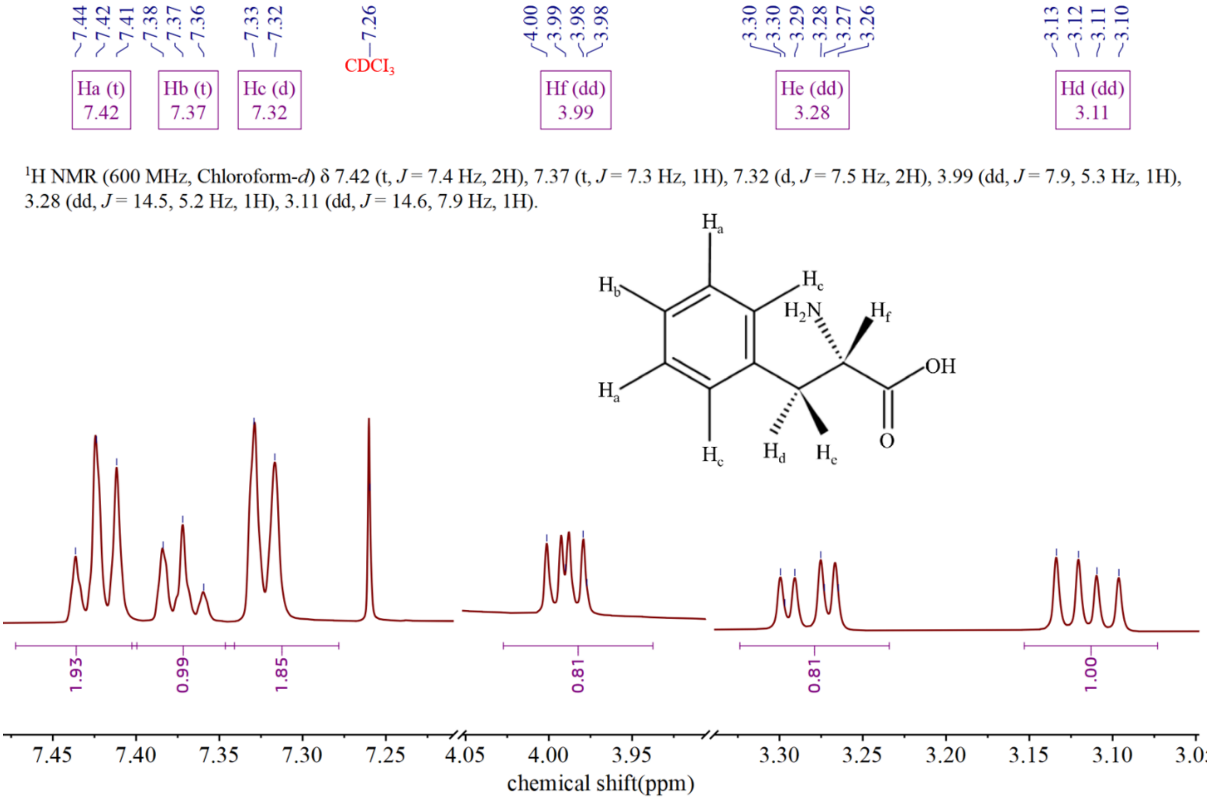

Figure S6 NMR profiles of L-Phe in bulk solution. ^{1}H (600 MHz; $CDCl_3$) NMR spectrum of L-phenylalanine. δ 7.42 (t, J = 7.4 Hz, 2H), 7.37 (t, J = 7.3 Hz, 1H), 7.32 (d, J = 7.5 Hz, 2H), 3.99 (dd, J = 7.9, 5.3 Hz, 1H), 3.28 (dd, J = 14.5, 5.2 Hz, 1H), 3.11 (dd, J = 14.6, 7.9 Hz, 1H).

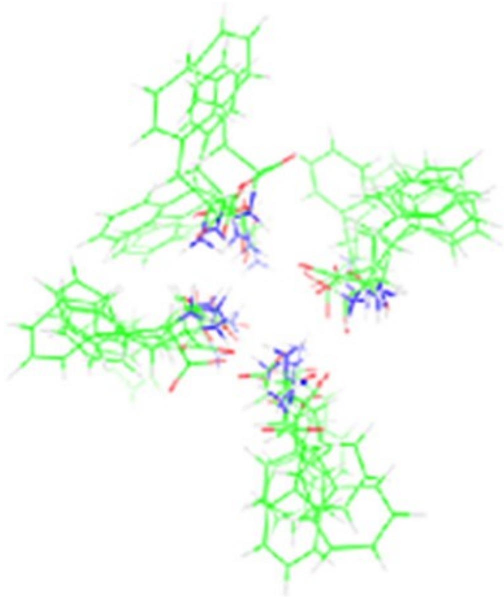

Figure S7 Proposed lowest energy structure of L-phenylalanine. Copyright © 2015, American Chemical Society.

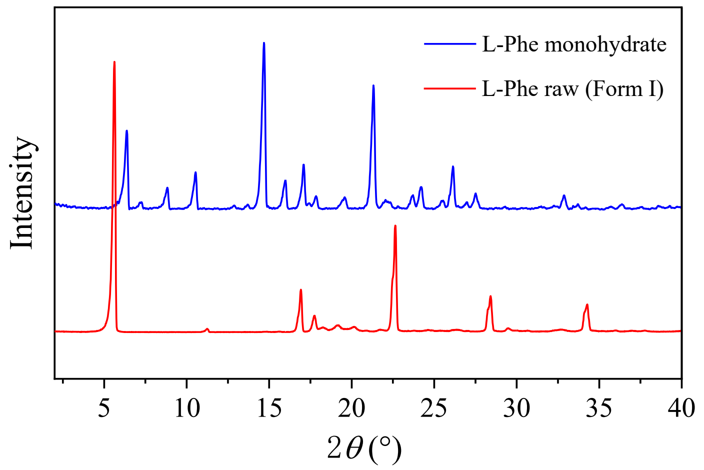

Figure S8 XRD of L-Phe. Red line is the prepared L-Phe monohydrate sample, blue line is raw of L-Phe (form I).
